# Evaluating the Accuracy of the Frysian Questionnaire for Differentiation of Musculoskeletal Complaints for Triage of Musculoskeletal Diseases: Algorithm Development and Validation Study

**DOI:** 10.2196/77345

**Published:** 2025-11-17

**Authors:** Tjardo Daniël Maarseveen, Floor Reimann, Ahmed al Hasan, Annemarie Schilder, Dan Zhang, Freke Wink, Lidy Hendriks, Rachel Knevel, Reinhard Bos

**Affiliations:** 1 Department of Rheumatology, Leiden University Medical Center, Albinusdreef 2, Leiden, 2333ZA, The Netherlands, 31 0715269111; 2 Department of Rheumatology, Frisius Medical CenterLeeuwarden, The Netherlands; 3 Department of Rheumatology, Newcastle University, Newcastle upon Tyne, United Kingdom

**Keywords:** medical informatics, questionnaire, machine learning, fibromyalgia, rheumatic diseases, rheumatoid arthritis, triage, decision support system, symptom checker

## Abstract

**Background:**

Inflammatory rheumatic diseases (IRDs) affect 5% of the general population, whereas 35% of the population experiences musculoskeletal concerns. IRDs cause early disability, reduced life expectancy, and considerable health care costs. Early diagnosis is essential to prevent long-term damage. Similarly important is the early identification of patients with musculoskeletal concerns without IRDs to prevent unnecessary health care expenses. Of the population referred to the rheumatologist, 60% have noninflammatory musculoskeletal concerns, whereas only 20% of patients with an IRD see a rheumatologist within 3 months of symptom onset. The need for digital predictive (triage) tools for rheumatic and musculoskeletal diseases led to the development of the Frysian Questionnaire for Differentiation of Musculoskeletal Complaints (FRYQ).

**Objective:**

This study aimed to assess whether the FRYQ can distinguish IRD from noninflammatory musculoskeletal concerns in general, and rheumatoid arthritis and fibromyalgia specifically, in newly referred patients.

**Methods:**

The FRYQ is an 87-item tool (20 open-ended and 67 closed-ended questions) used to triage new rheumatology patients at Frisius Medical Center in the Netherlands. We analyzed data from 2 sources: dataset A with 728 outpatient clinic patients and dataset B with 373 patients from the Joint Pain Assessment Scoring Tool study. We built a classifier using Extreme Gradient Boosting to distinguish inflammatory from noninflammatory conditions based on closed-ended questions. Using elastic net regularization, we identified the most informative questions. We evaluated classification using receiver operating characteristic curve analysis and assessed feature importance through Shapley Additive Explanation analysis. To test generalizability, we replicated our analysis on dataset B. Finally, we examined whether the questions of the FRYQ could be used to identify specific conditions beyond the general categories of IRD and non-IRD, specifically for detecting fibromyalgia and rheumatoid arthritis.

**Results:**

Feature selection reduced the questionnaire from 67 to 28 items while maintaining discriminative power. After initial development, the model achieved an area under the receiver operating characteristic curve (AUC-ROC) of 0.72 (95% CI 0.67-0.78) for distinguishing inflammatory from noninflammatory conditions in an external validation set. Using a probability threshold of 0.30, the model achieved 71% sensitivity and 56% specificity on external validation. The FRYQ demonstrated stronger performance in identifying specific diagnoses such as fibromyalgia (AUC-ROC=0.81) and rheumatoid arthritis (AUC-ROC=0.77). Key discriminating features included symptom duration, pain response to movement, and anti-inflammatory medication effectiveness.

**Conclusions:**

The FRYQ effectively distinguishes inflammatory from noninflammatory rheumatic conditions before specialist consultation and shows particular strength in identifying fibromyalgia and rheumatoid arthritis. This tool could improve rheumatology triage by prioritizing referrals with high likelihood of IRD for early rheumatologist evaluation while directing other patients to appropriate alternative resources. Prospective studies are needed to determine the FRYQ’s impact on clinical outcomes and health care efficiency.

## Introduction

Inflammatory rheumatic diseases (IRDs) affect 5% of the general population in the Netherlands, whereas 35% of the population experience musculoskeletal concerns [1]. Global prevalence of both IRDs and musculoskeletal concerns varies with age and region but is comparable to that in the Netherlands [2]. IRDs can cause early disability, reduced life expectancy, and considerable health care costs.

In the Dutch health care system, as in many European countries, patients with musculoskeletal concerns first consult a general practitioner (GP) before referral to a specialist. To facilitate appropriate and timely care, the Dutch College of General Practitioners (*Nederlands Huisartsen Genootschap*) has developed national guidelines and referral protocols [3], helping ensure that patients receive specialist attention when necessary.

Early diagnosis of rheumatic diseases such as rheumatoid arthritis (RA), ankylosing spondylitis, or psoriatic arthritis by specialists is essential to prevent irreversible damage [4-7]. Similarly important is the early identification of patients with musculoskeletal concerns without IRDs who can be treated in primary care to prevent unnecessary health care expenses. This need is particularly pressing in the Netherlands, which has one of the most expensive health care systems in Europe—ranking third after Austria and Germany [8].

Approximately 109 new patients with musculoskeletal concerns per 1000 people are seen by GPs annually [9]. Of the population referred to a rheumatologist, 60% have noninflammatory musculoskeletal concerns, whereas only 20% of patients with an IRD see a rheumatologist within 3 months of symptom onset [10]. For the target disease, RA, we observe that these patients have to wait—on average—4 weeks from referral to their visit to a rheumatologist [11].

The urgent need for more efficient management of referred patients led to the development of the Frysian Questionnaire for Differentiation of Musculoskeletal Complaints (FRYQ). The FRYQ is an 87-item questionnaire consisting of 20 open-ended and 67 close-ended questions that is used in regular care in the rheumatology outpatient clinic of Frisius Medical Center to triage newly referred patients. It was developed to improve diagnostic speed for referred patients by facilitating early appropriate blood testing.

Our study had three main objectives: (1) evaluate the FRYQ’s ability to differentiate IRDs from non-IRDs in newly referred patients using machine learning techniques; (2) identify which questions are most informative for distinguishing inflammatory conditions and develop a streamlined version without losing diagnostic value; and (3) assess whether the FRYQ can be used to identify specific conditions of interest, particularly fibromyalgia and RA, which represent important diagnostic targets in rheumatology practice.

## Methods

### Study Design and Source of Data

**Figure 1.**
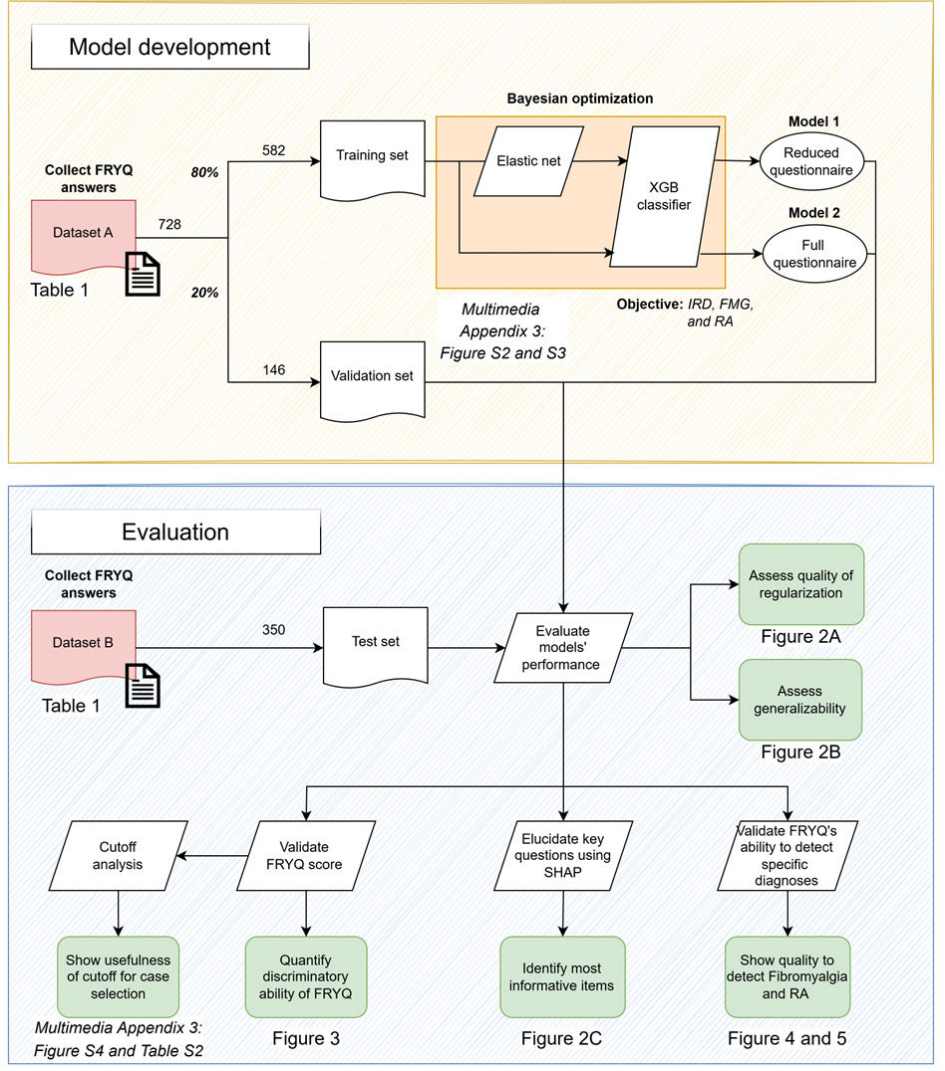
Study workflow consisting of two phases: (1) model development, where we built a classifier to detect inflammatory rheumatic diseases (IRDs), fibromyalgia, and rheumatoid arthritis (RA) using only Frysian Questionnaire for Differentiation of Musculoskeletal Complaints (FRYQ) responses+sex based on the data from the first pilot (n=728), and (2) model evaluation, where we deployed the model on unseen data from a different study (n=350) to assess generalizability. FMG: fibromyalgia; SHAP: Shapley Additive Explanations; XGB: Extreme Gradient Boosting.

In this study, we conducted a retrospective analysis of newly referred patients to the rheumatology department at Frisius Medical Center, Leeuwarden, the Netherlands (the pipeline is shown in Figure 1). We developed and validated machine learning prediction models to assess the FRYQ’s ability to differentiate inflammatory from noninflammatory rheumatic conditions using only preconsultation patient responses. Multimedia Appendix 1 provides the Dutch version of the questionnaire, and Multimedia Appendix 2 provides the translated version in English.

We used two independent cohorts: (1) a development dataset (dataset A), which comprised 728 adult patients who completed the FRYQ between August 2017 and June 2019 as part of routine care, and (2) an external validation dataset (dataset B), which comprised 373 adult patients from the Joint Pain Assessment Scoring Tool (JPAST) study cohort [12] visiting the clinic between January 2023 and March 2024 who completed the FRYQ as an additional research instrument.

### Ethical Considerations

The FRYQ was developed by the rheumatology clinic at the Frisius Medical Center to assist in prioritizing newly referred patients. The data in dataset A were gathered as part of standard clinical care. Informed consent for the use of anonymized data in research was secured through the opt-out procedure, whereby all patient data were eligible for inclusion unless an individual filed a formal objection. According to Article 1(b) of the Dutch Medical Research Involving Human Subjects Act (Wet medisch-wetenschappelijk onderzoek met mensen [WMO]), ethical review is required only for studies that aim to obtain medical-scientific knowledge and in which participants are subjected to an intervention or are required to follow a behavioral regime [13]. As the FRYQ questionnaire was administered as part of routine care and did not involve any intervention or imposed behavior, the study does not fall under the scope of the WMO and therefore did not require approval by a medical ethics review committee.

In contrast, dataset B consisted of patients enrolled in the JPAST study, which involved time-consuming study procedures. Therefore, the use of the FRYQ in this dataset was reviewed and approved by the medical ethics committee of the Frisius Medical Center (reference number RTPO-1104) as part of the JPAST study protocol. Since participation in the JPAST study involved procedures beyond standard care, each participant provided written informed consent authorizing the use of their data for research.

For both datasets, patient data security was ensured by assigning each patient a unique sequential identifier through pseudonymization. Data were stored and analyzed in a secure, password-protected database accessible only to authorized personnel. Participants retained the right to opt out of the use of their data for research purposes and did not receive financial compensation for their involvement in this study. All study procedures were conducted in accordance with the principles of the Declaration of Helsinki.

### Participants

#### Eligibility Criteria

All adult patients newly referred to the rheumatology outpatient clinic who completed the FRYQ were eligible. From dataset A, 854 patients were initially invited, with 728 (85.2%) having complete data for analysis. From dataset B, all patients with complete FRYQ responses and diagnosis data were included.

#### Study Setting

Data collection took place in a nonacademic hospital setting (Frisius Medical Center) during routine clinical care for dataset A and as part of the international JPAST validation study for dataset B.

### Outcome

#### Primary Outcome

The primary outcome was the presence of an IRD versus a noninflammatory musculoskeletal condition (non-IRD) as diagnosed by a rheumatologist after clinical evaluation. This determination represented the clinical ground truth against which the model predictions were evaluated. The outcome was assessed by extracting the final diagnosis from electronic health records.

#### Secondary Outcomes

Specific diagnoses of fibromyalgia and RA were evaluated as secondary outcomes in additional predictive models.

### Predictors (Input Variables)

The primary input variables consisted of responses to the 67 closed-ended questions from the FRYQ supplemented by patient age and sex. The FRYQ is an 87-item tool (including 20 open-ended questions not used in this analysis) designed by rheumatologists for triaging rheumatology patients. Question topics covered pain characteristics and patterns, stiffness symptoms and duration, functional limitations, associated symptoms, comorbid conditions, medication responses, and patient behaviors and characteristics. No laboratory test results or imaging data were included as predictors in the model.

### Missing Data

Patients with incomplete demographic and questionnaire data were removed from dataset A (127/862, 14.7%) and dataset B (166/552, 30.1%; Figure S1 in Multimedia Appendix 3). Furthermore, we had some patients with inconsistent diagnostic labels removed from dataset A (7/862, 0.8%) and a few with missing diagnosis information removed from dataset B (36/552, 6.5%).

### Model Development

#### Data Preprocessing

No significant preprocessing was required as the questionnaire items were already structured. Patient sex was included as a binary variable, and patient age was included as a continuous variable.

#### Feature Selection

We used elastic net regularization to identify the most informative subset of questions from the original 67 closed-ended items. The regularization parameters (alpha and L1 ratio) were optimized using Bayesian methods with a tree-structured Parzen estimator across 1000 iterations [14]. This approach allowed us to reduce dimensionality while retaining predictive power.

#### Model Specification

We used the Extreme Gradient Boosting technique [15] to build a model for IRD prediction using only the questions selected by the elastic net. Extreme Gradient Boosting was chosen for its ability to capture complex relationships between variables and its robust performance in previous prediction tasks [16]. All machine learning analyses were performed in Python (version 3.6.13; Python Software Foundation).

#### Model Training

The model was trained on 80% of dataset A. We used Bayesian optimization across 1000 iterations to tune model hyperparameters, optimizing for root mean square logarithmic error to emphasize sensitivity to inflammatory conditions.

### Model Performance

#### Measures of Model Performance

We evaluated model discrimination using the area under the receiver operating characteristic curve (AUC-ROC). Calibration was assessed using calibration plots comparing predicted probabilities with observed outcome frequencies.

#### Validation

The model was first validated on the reserved 20% of dataset A (internal validation) and then on dataset B (external validation) to assess generalizability. Both the reduced model (with selected features) and the full model (with all 67 questions) were evaluated to assess the impact of feature selection.

### Clinical Utility

We conducted threshold analysis to identify an optimal probability cutoff for distinguishing IRD from non-IRD cases in clinical practice, evaluating sensitivity, specificity, positive predictive value, and negative predictive value at various thresholds.

### Model Interpretation

To understand feature importance and explain model predictions, we conducted Shapley additive explanation (SHAP) analysis [17]. This approach quantified the contribution of each question to the model output, allowing us to identify which questions were most informative for detecting inflammatory conditions.

## Results

### Overview

Dataset A comprised 728 patients, of whom 239 (32.8%) were male and 489 (67.2%) were female (Table 1). Nearly half (257/582, 44.2%) of the patients in the training dataset had IRDs. The most common disorders associated with IRD were RA (62/582, 10.7%), gout (45/582, 7.7%), and spondyloarthritis (43/582, 7.4%). Among noninflammatory arthropathies, the most frequent were osteoarthritis (119/582, 20.4%), tendinopathy (92/582, 15.8%), and fibromyalgia (91/582, 15.6%).

The external test dataset B included 350 patients with complete information, comprising 106 (30.3%) male and 244 (69.7%) female individuals. Only 26.6% (93/350) of the patients were diagnosed with an IRD, which was notably lower than those in the training and validation partitions of dataset A (257/582, 44.2% and 67/146, 45.9%, respectively). In contrast, the JPAST data contained a higher proportion of patients with an “other” diagnosis, indicating that this dataset represented a somewhat different patient population.

**Table 1.** Patient characteristics in both datasets.

	Dataset A		Dataset B
	Training (n=582)	Hold-out (n=146)	Replication (n=350)
Sex (female), n (%)	308 (52.9)	97 (66.4)	244 (69.7)
Age (y), mean (SD)	52 (17)	55 (17)	53 (16)
IRD^a^, n (%)	257 (44.2)	67 (45.9)	93 (26.6)
Rheumatoid arthritis	62 (10.7)	25 (17.1)	15 (4.3)
Polymyalgia rheumatica	20 (3.4)	7 (4.8)	12 (3.4)
Gout	45 (7.7)	4 (2.7)	11 (3.1)
Systemic lupus erythematosus	3 (0.5)	2 (1.4)	0 (0)
Psoriatic arthritis	20 (3.4)	9 (6.2)	4 (1.1)
Spondyloarthritis	43 (7.4)	9 (6.2)	13 (3.7)
Sjögrenn disease	5 (0.9)	1 (0.7)	1 (0.3)
Reactive arthritis	9 (1.5)	3 (2.1)	2 (0.6)
Undifferentiated arthritis	9 (1.5)	1 (0.7)	14 (4)
Systemic sclerosis	4 (0.7)	1 (0.7)	1 (0.3)
CPPD^b^	10 (1.7)	3 (2.1)	2 (0.6)
Probable RMD^c^ in development	17 (2.9)	1 (0.7)	18 (5.1)
Sarcoidosis	9 (1.5)	1 (0.7)	0 (0)
ANCA^d^-associated vasculitis	1 (0.2)	0 (0)	0 (0)
No IRD, n (%)	323 (55.5)	79 (54.1)	185 (52.9)
Osteoarthritis	119 (20.4)	33 (22.6)	73 (20.9)
Tendinopathy	92 (15.8)	25 (17.1)	39 (11.1)
Fibromyalgia	91 (15.6)	19 (13)	68 (19.4)
Pernio	0 (0)	0 (0)	4 (1.1)
Hypermobility	21 (3.6)	2 (1.4)	1 (0.3)
Ambiguous, n (%)	2 (0.3)	0 (0)	72 (20.6)
Other	0 (0)	0 (0)	65 (18.6)
Unknown	2 (0.3)	0 (0)	7 (2)

a IRD: inflammatory rheumatic disease.

b CPPD: calcium pyrophosphate crystal deposition disease.

c RMD: rheumatic and musculoskeletal disease.

d ANCA: antineutrophil cytoplasmic antibody.

### Prediction of IRD

After hyperparameter optimization (α=.07; L1-ratio=0.16; Figures S2 and S3 in Multimedia Appendix 3) and feature selection, our model reduced the 67 closed-ended questions to 28 key items (Table S1 in Multimedia Appendix 3). This streamlined model effectively distinguished IRD from non-IRD across multiple validation steps.

**Figure 2.**
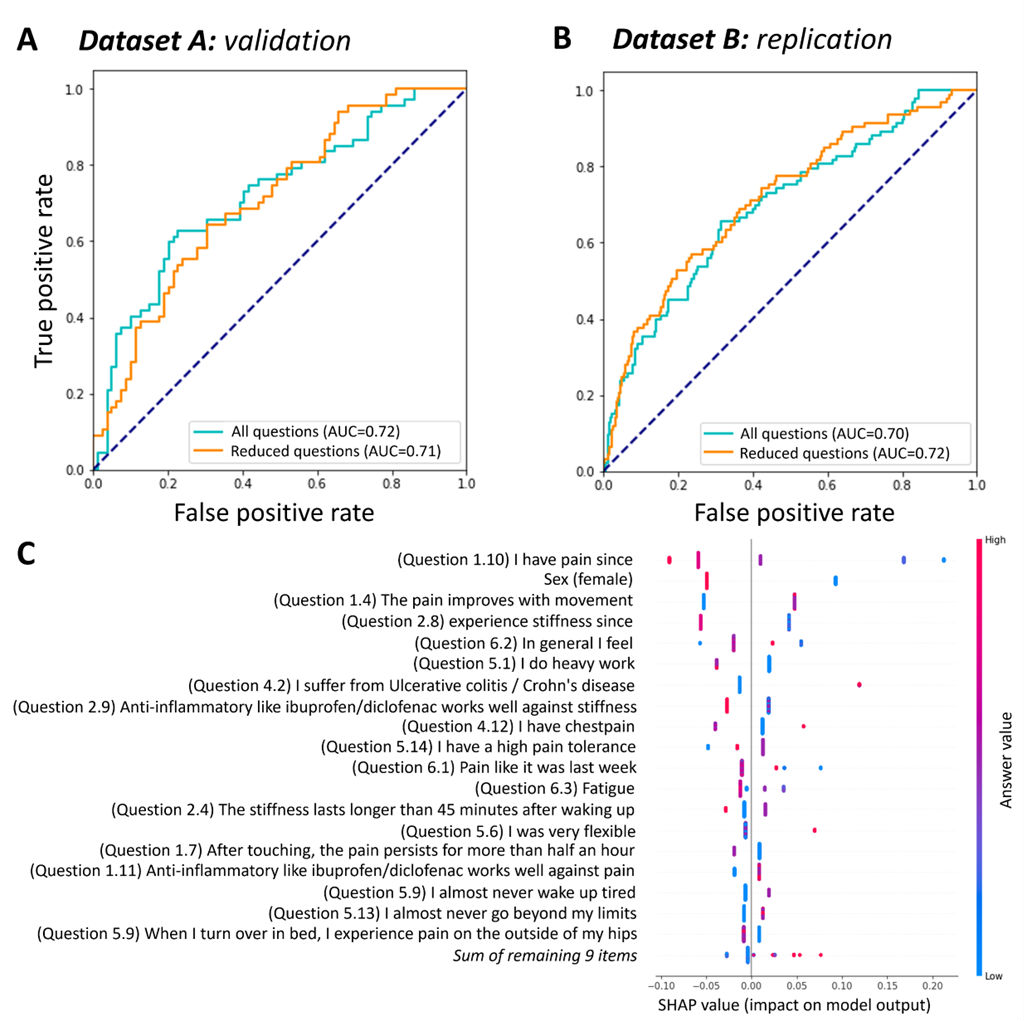
Performance of the inflammatory rheumatic disease classifier: (A) receiver operating characteristic (ROC) curves showing performance before and after regularization in the validation set, (B) ROC curves showing performance before and after regularization in the replication set, and (C) Shapley Additive Explanation (SHAP) analysis results highlighting the impact and direction of the most important features contributing to the classifier’s predictions. AUC: area under the curve.

In the development dataset A, the reduced model achieved an AUC-ROC of 0.70 (95% CI 0.67-0.74) in the training set and 0.71 (95% CI 0.64-0.78) in the hold-out validation set, performing comparably to the full model (AUC-ROC of 0.67, 95% CI 0.63-0.72 and 0.72, 95% CI 0.64-0.78, respectively; Figure 2A).

Importantly, when tested in the independent dataset B, the model demonstrated robust generalizability, with an AUC-ROC of 0.72 (95% CI 0.67-0.78) for the reduced model and 0.68 (95% CI 0.63-0.75) for the full model (Figure 2B), confirming the model’s stability across different patient populations.

**Figure 3.**
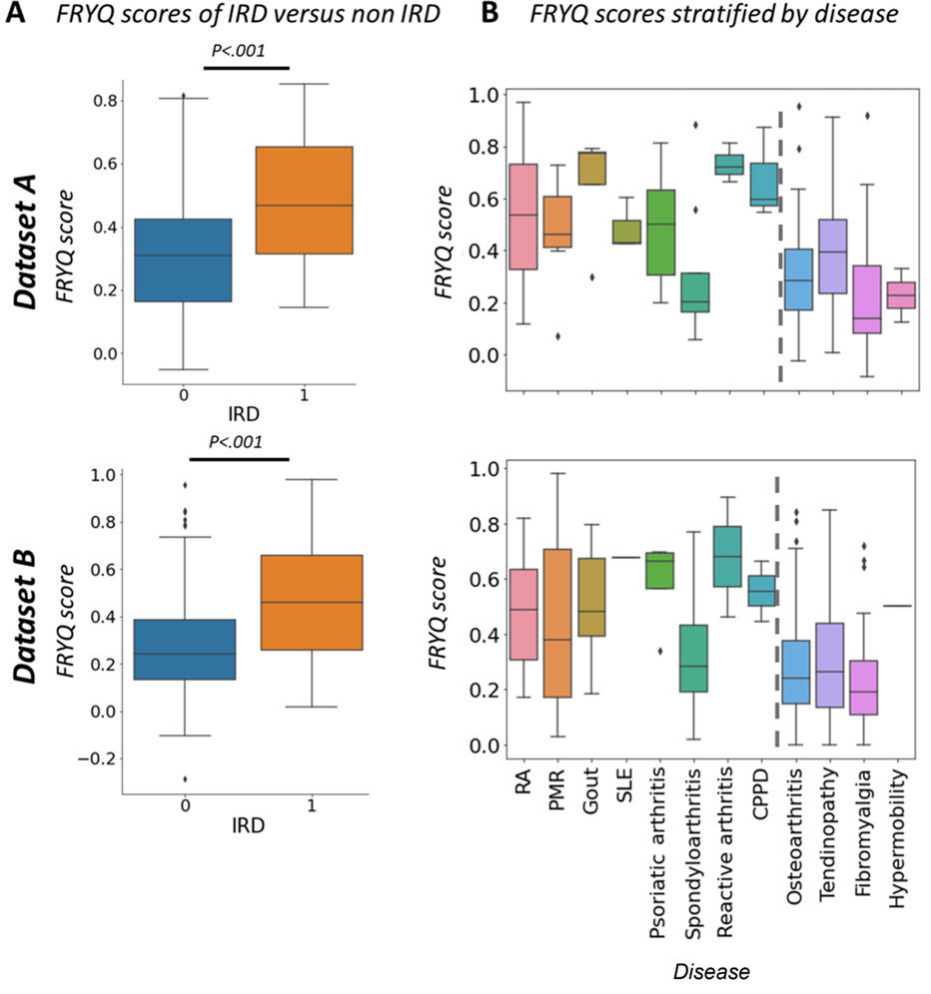
Overview of Frysian Questionnaire for Differentiation of Musculoskeletal Complaints (FRYQ) score distributions (A) between inflammatory rheumatic disease (IRD) and non-IRD and (B) more specifically by disease, with IRDs on the left of the dashed line and non-IRDs on the right. The comparison between IRDs and non-IRDs was conducted using a 2-tailed Student *t* test. CPPD: calcium pyrophosphate crystal deposition disease (pseudogout); PMR: polymyalgia rheumatica; RA: rheumatoid arthritis; SLE: systemic lupus erythematosus. *P<.05; **P<.01; ***P<.001.

The FRYQ score (model-predicted probability) consistently distinguished IRD patients from non-IRD patients across both datasets (*P*<.001; Figure 3). In the development set A, the median FRYQ score was 0.47 (IQR 0.31-0.65) for patients with IRD, versus 0.30 (IQR 0.16-0.42) for patients without IRD. Similarly, in replication set B, the median score was 0.46 (IQR 0.26-0.66) for patients with IRD, compared to 0.24 (IQR 0.13-0.39) for patients without IRD. When examining specific conditions, most IRDs exhibited higher scores than non-IRDs, with 2 notable exceptions: spondyloarthritis consistently scored lower among IRDs, whereas tendinopathy scored relatively high (>0.35) among non-IRDs, suggesting areas where diagnostic discrimination was more challenging. Fibromyalgia consistently showed very low scores (<0.2), making it a promising target for specialized detection.

The key features for identifying an IRD in the newly referred population (Figure 2C) were questions related to duration of concerns (questions 1.10 and 2.8), male sex, and decrease of pain upon movement (question 1.4). Moreover, the presence of certain comorbidities influenced the model’s assessment. Ulcerative colitis and Crohn disease (question 4.2) showed a positive association with IRDs, whereas chest pain (question 4.12) showed a negative association. Additionally, respondents diagnosed with an IRD more frequently reported that nonsteroidal anti‐inflammatory drugs such as ibuprofen and diclofenac were effective in managing their symptoms (questions 2.9 and 1.11).

Next, we defined the optimal clinical threshold for case selection. On the basis of the training data, we set the cutoff at 0.30 (Figure S4 in Multimedia Appendix 3) as it captured more than three-fourths of IRD cases (sensitivity=0.76) while ensuring that half of the cases without IRDs were excluded (specificity=0.49). This threshold showed similar sensitivity (0.71) and specificity (0.56) in the external validation dataset. The positive predictive value was lower in the external validation dataset (0.39 vs 0.56 in dataset A), attributable to the lower IRD prevalence (93/350, 26.6% vs 257/582, 44.2%; Table S4 in Multimedia Appendix 3).

The calibration curve indicated that the model slightly overestimated IRD probability in the external test dataset (Figure S5 in Multimedia Appendix 3), reflecting the difference in disease prevalence between the training and validation populations. Despite this, the discriminatory ability remained strong, with IRDs consistently receiving higher scores than non-IRDs across both datasets, reinforcing the FRYQ’s potential value as a triage tool.

### Prediction of Specific Diagnoses: Fibromyalgia and RA

Our analysis extended beyond distinguishing inflammatory from noninflammatory conditions to evaluate the FRYQ’s ability to identify specific diagnoses. Patients with fibromyalgia showed particularly distinctive response patterns compared to those with inflammatory conditions (Figure 3), making this a compelling diagnostic target with sufficient representation in our datasets.

**Figure 4.**
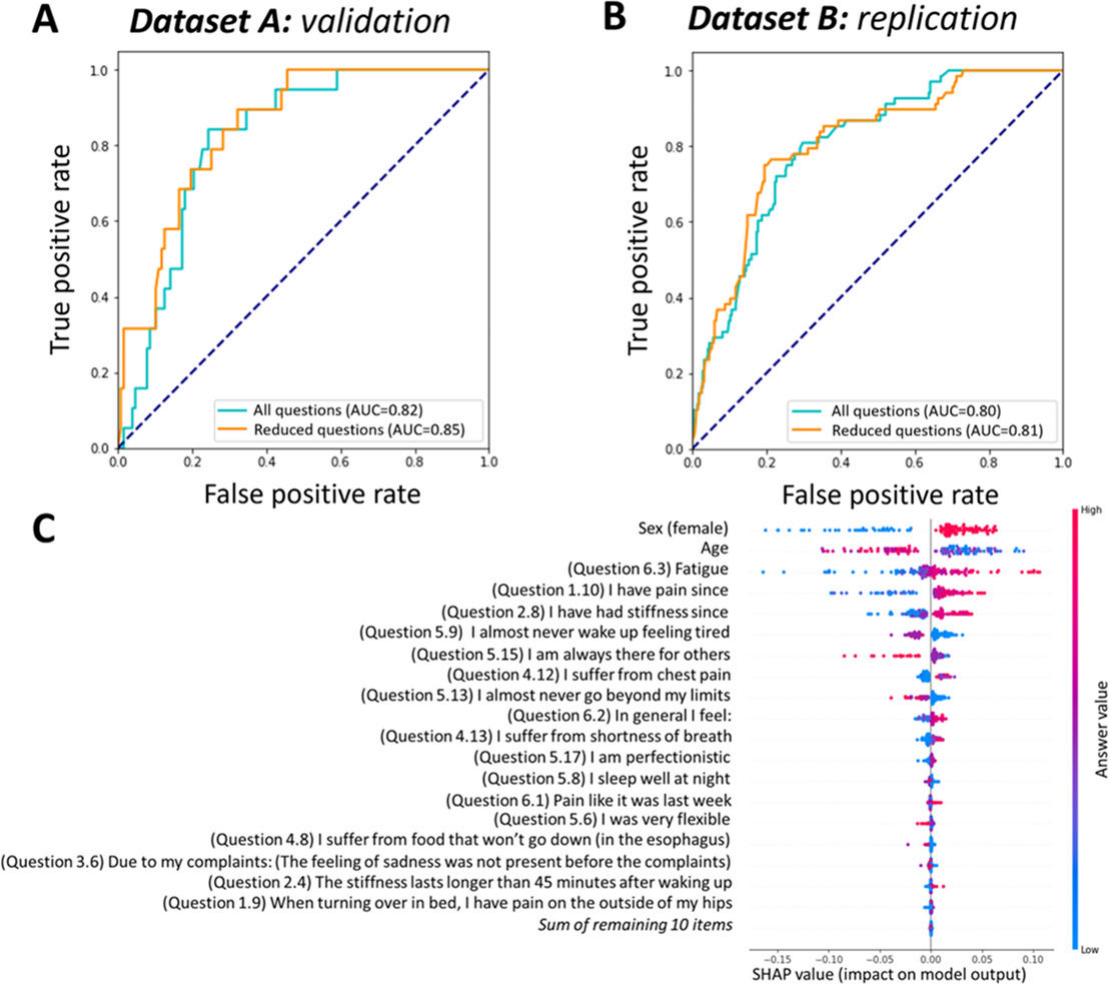
Performance of the fibromyalgia classifier: (A) receiver operating characteristic (ROC) curves showing performance before and after regularization in the validation set, (B) ROC curves showing performance before and after regularization in the replication set, and (C) Shapley Additive Explanation (SHAP) analysis results highlighting the impact and direction of the most important features contributing to the classifier’s predictions. AUC: area under the curve.

The fibromyalgia-specific model demonstrated strong discriminative ability, achieving an AUC-ROC of 0.85 (95% CI 0.78-0.90) in the development dataset and maintaining robust performance in the external validation dataset with an AUC-ROC of 0.81 (95% CI 0.75-0.86; Figure 4). SHAP analysis identified key predictive features for fibromyalgia: female sex, younger to middle age, fatigue (question 6.3), chest pain (question 4.12), extended symptom duration (questions 1.10 and 2.8), and waking up tired (question 5.9). The model effectively differentiated fibromyalgia from RA, although some misclassification occurred with noninflammatory conditions such as hypermobility (Figure 6A in Multimedia Appendix 3).

**Figure 5.**
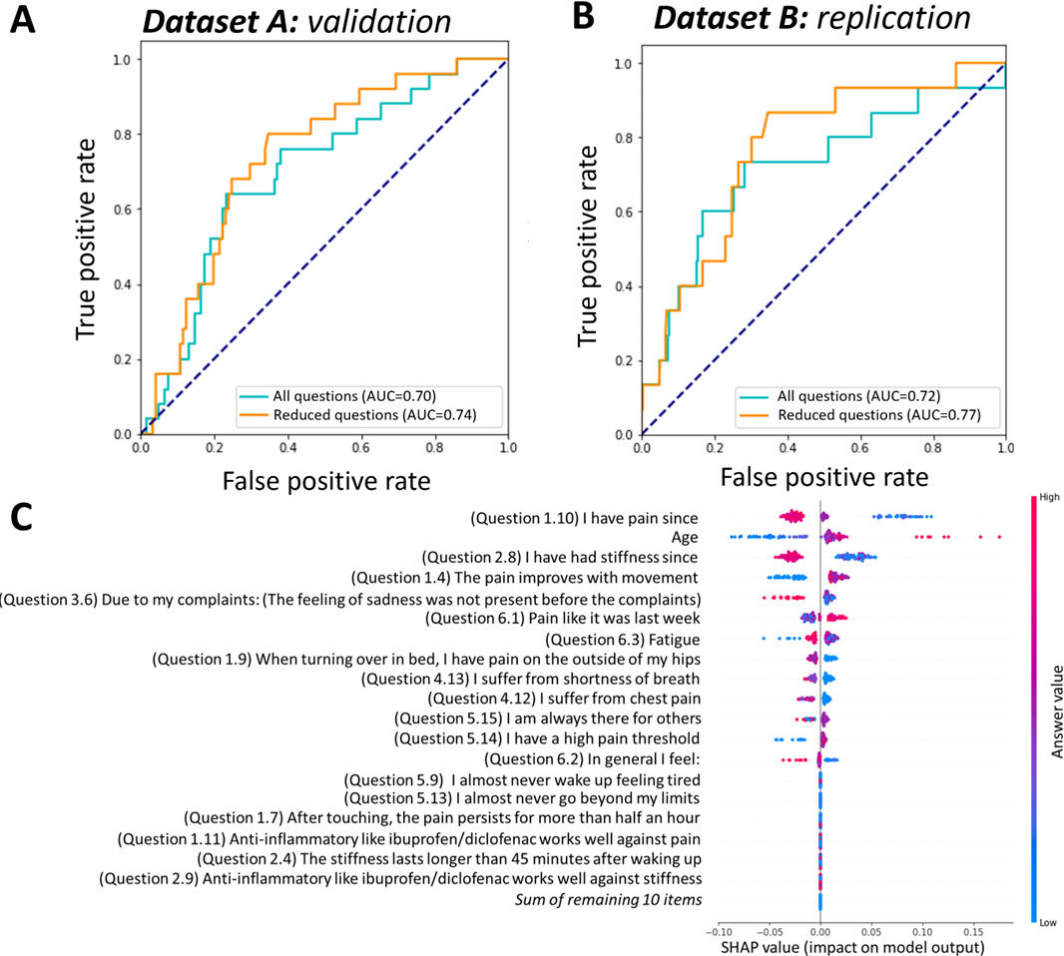
Performance of the rheumatoid arthritis classifier: (A) receiver operating characteristic (ROC) curves showing performance before and after regularization in the validation set, (B) ROC curves showing performance before and after regularization in the replication set, and (C) Shapley Additive Explanation (SHAP) analysis results highlighting the impact and direction of the most important features contributing to the classifier’s predictions. AUC: area under the curve.

For RA—a critical target for early rheumatology intervention—our model showed moderate discrimination, with AUC-ROC values of 0.74 (95% CI 0.63-0.83) in the development set and 0.77 (95% CI 0.66-0.86) in the external validation set (Figure 5). Key predictors included shorter disease duration (questions 1.10 and 2.8), pain improvement with movement (question 1.4), recent severe pain (question 6.1), onset at middle age or later, and fewer respiratory or chest symptoms (questions 4.13 and 4.12) or hip concerns (question 1.9). The model’s primary challenge was differentiating RA from other IRDs with similar presentations, such as reactive arthritis, polymyalgia rheumatica, and gout (Figure 6B in Multimedia Appendix 3).

## Discussion

### Principal Findings

Our results demonstrate that the FRYQ can effectively distinguish between IRDs and noninflammatory musculoskeletal concerns before patients’ first rheumatology consultation. The model achieved robust performance, with an AUC-ROC of 0.71 in the development dataset and 0.72 in the external validation dataset, indicating good generalizability. Importantly, we found that a reduced set of just 28 questions from the original 67 closed-ended items maintained equivalent discriminative power, suggesting potential for a more efficient screening tool.

Beyond the primary classification task, our analysis revealed that the FRYQ demonstrates even stronger performance in identifying specific conditions, particularly fibromyalgia (AUC-ROC of 0.85 and 0.81 in the development and replication datasets, respectively). The FRYQ also showed adequate ability to detect RA cases (AUC-ROC of 0.77 in the external validation dataset), although it had more difficulty distinguishing RA from other inflammatory conditions.

These findings have important clinical applications. In settings with limited rheumatology resources, the FRYQ could serve as a triage tool to streamline the large influx of referrals (overreferral) by prioritizing the referred patients with higher probability of inflammatory disease for early specialist intervention. This could result in more efficient allocation of specialist resources, potentially reducing waiting lists for a substantial number of patients with IRD. In light of the anticipated shortage of clinical professionals, it is increasingly important to identify which patients truly need rheumatologists’ care versus those who can be managed elsewhere [18].

For example, the FRYQ’s ability to identify fibromyalgia among the rheumatology waiting list could help redirect these patients to alternative care providers instead (pain specialists, physiotherapists, or dedicated programs), freeing capacity at the clinic for patients needing rheumatological care. Currently, rheumatologists spend substantial time on patients they cannot effectively treat as approximately 60% of rheumatology referrals involve noninflammatory conditions (such as fibromyalgia) [10]. This optimized allocation may also offer an additional benefit: the extra capacity created could reduce primary care physicians’ reluctance to refer clinically ambiguous cases that would benefit from specialist input.

Finally, the FRYQ could aid clinicians in their decision to request extra laboratory workup. When responses suggest RA, clinicians could order relevant serological markers (anticitrullinated protein antibody, rheumatoid factor, C-reactive protein, and erythrocyte sedimentation rate) before the initial visit, potentially streamlining the diagnostic process and reducing time to treatment for conditions requiring early intervention [4-7].

The clinical threshold of 0.30 that we identified balances sensitivity (capturing 71%-76% of inflammatory cases) with reasonable specificity (49%-56%), making it suitable for initial screening where missing IRD cases would be more concerning than false positives. As reported by Graydon and Thompson [19], primary care physicians and rheumatologists frequently disagree on referral urgency (47%), with rheumatologists upgrading 17% of referrals to urgent status—cases initially overlooked by GPs. This underlines the potential value of applying such a threshold in practice, although adjustments may be required depending on local context, such as disease prevalence and health care setting.

Our findings align with the growing recognition that structured questionnaires can support diagnostic decision-making in rheumatology by systematically capturing and integrating patients’ own experiences and symptom descriptions. While over 100 symptom checkers currently exist [20], their adoption has remained limited due to lack of validation or suboptimal diagnostic accuracy [20,21]. Therefore, the availability of a validated tool such as the FRYQ may fulfill a need in the field. As far as we know, this is the first questionnaire developed in the Netherlands for triaging musculoskeletal concerns that has been validated using real-world patient data.

The predictive factors identified in our SHAP analysis align with established clinical knowledge. Pain improvement with movement, shorter morning stiffness duration, and good response to nonsteroidal anti-inflammatory drugs all emerged as important indicators of inflammatory disease [22-24]. The association between inflammatory bowel disease and IRDs supports known connections between these conditions [25]. For fibromyalgia, we observe that patients report sleep problems, persistent pain, and fatigue [26].

Beyond questionnaires, there have been other digital approaches proposed in the literature for improving rheumatology triage. Natural language processing (NLP) of referral letters has shown promising results in studies from the United Kingdom [27] and the Netherlands [28], potentially complementing questionnaire-based methods. Similarly, patient-reported outcomes have been investigated, although Schäfer et al [29] found these to be less effective at distinguishing inflammatory from noninflammatory rheumatic conditions.

### Study Limitations

Several limitations of this study should be acknowledged. First, while we validated our model in an external dataset, both cohorts originated from the same medical center, potentially limiting generalizability to different health care settings or patient populations. Second, we observed a rather big difference in prevalence of inflammatory conditions (257/582, 44.2% vs 93/350, 26.6%). This affected the model’s calibration in external validation, suggesting the need for recalibration when applying to populations with different disease prevalence.

These prevalence differences likely stem from distinct patient selection mechanisms. Dataset A included patients with prescheduled outpatient appointments who completed the questionnaire during their first visit as part of routine care in Medical Center Leeuwarden (approximately 70% of referrals). In contrast, dataset B comprised volunteers from the JPAST study (approximately 40% of referrals). The time-consuming study procedures of the JPAST study likely led to an overrepresentation of nonurgent (non-IRD) cases among the volunteers.

We should also acknowledge that our model showed moderate discriminative ability (AUC of approximately 0.72) rather than excellent performance. Therefore, it could not reliably distinguish certain conditions such as spondyloarthritis from noninflammatory diseases. Rare inflammatory conditions were also underrepresented.

Crucially, our study was tailored to patients already referred to rheumatologists with the goal of optimizing the time from referral to specialist consultation. This means that the FRYQ cannot be used as a prescreening tool at GP offices. Importantly, it was not designed to exclude patients from care but rather to guide referred patients to the most appropriate care providers and streamline referral pathways.

### Future Directions

To definitively establish the FRYQ’s clinical utility, we propose conducting a randomized clinical trial in the future to assess whether FRYQ-guided triage leads to faster appropriate treatment, reduced unnecessary appointments, and improved patient satisfaction. Furthermore, our study would benefit from additional replication in other countries to assess how health care system differences affect its effectiveness. Referral rates and treatment protocols vary substantially between countries—for instance, 21% of patients with musculoskeletal conditions are referred to specialists in Spain compared to 11% in the Netherlands and 5% in Sweden [30].

In the future, we also plan to incorporate NLP techniques to leverage open-ended questions as they may capture nuanced symptom descriptions that structured items tend to miss. Modern NLP methods, including large language models, show potential for supporting triage [31] and the identification of rare or unrecognized diseases using free-text data [32,33]. For spondyloarthritis in particular, large language models could be prompted with educational information on axial spondyloarthritis recognition to improve detection.

Finally, we may enhance the questionnaire by incorporating questions on occupational stress given that it is a known predictor of non-IRD [34,35]. Further refinement could also involve integrating FRYQ scores with serological markers and imaging to create a comprehensive multimodal prediction model. This multimodal approach might overcome some limitations of questionnaire-based triage alone, particularly for discerning conditions with overlapping symptomatology (ie, overlapping symptoms of osteoarthritis and early RA [36]).

### Conclusions

The FRYQ shows promise in distinguishing inflammatory from noninflammatory rheumatic conditions in newly referred patients, particularly fibromyalgia and RA, before specialist assessment. While not a substitute for clinical judgment, this tool could help optimize the time from referral to specialist consultation by prioritizing cases likely to involve IRDs and guiding others toward alternative care pathways or additional laboratory testing. These potential benefits merit evaluation in prospective implementation studies, especially given the projected shortage of rheumatology professionals.

## Supplementary material

10.2196/77345 Multimedia Appendix 1 The original Frysian Questionnaire for Differentiation of Musculoskeletal Complaints in Dutch.

10.2196/77345 Multimedia Appendix 2 Table comprising the English translation of all original Dutch questions from the Frysian Questionnaire for Differentiation of Musculoskeletal Complaints.

10.2196/77345 Multimedia Appendix 3 Supplementary results supporting the main analysis, including patient selection details, model optimization procedures, comprehensive performance metrics and calibration analyses, the final questionnaire items selected by the model, and results from disease-specific classification models.

## Abbreviations

AUC-ROC: area under the receiver operating characteristic curve
FRYQ: Frysian Questionnaire for Differentiation of Musculoskeletal Complaints
GP: general practitioner
IRD: inflammatory rheumatic disease
JPAST: Joint Pain Assessment Scoring Tool
NLP: natural language processing
RA: rheumatoid arthritis
SHAP: Shapley Additive Explanations
WMO: Wet medisch-wetenschappelijk onderzoek met mensen

## References

[1] van der Linden MW, Westert GP, Schellevis F (2004). Tweede nationale studie naar ziekten en verrichtingen in de huisartspraktijk: klachten en aandoeningen in de bevolking en in de huisartspraktijk [Report in Dutch]. https://www.nivel.nl/sites/default/files/bestanden/ns2_r1_h02.pdf.

[2] GBD 2019 Diseases and Injuries Collaborators (2020). Global burden of 369 diseases and injuries in 204 countries and territories, 1990-2019: a systematic analysis for the global burden of disease study 2019. Lancet.

[3] (2023). NHG-richtlijnen [Web page in Dutch]. Nederlands Huisartsen Genootschap.

[4] Saalfeld W, Mixon AM, Zelie J, Lydon EJ (2021). Differentiating psoriatic arthritis from osteoarthritis and rheumatoid arthritis: a narrative review and guide for advanced practice providers. Rheumatol Ther.

[5] Nell VPK, Machold KP, Eberl G, Stamm TA, Uffmann M, Smolen JS (2004). Benefit of very early referral and very early therapy with disease-modifying anti-rheumatic drugs in patients with early rheumatoid arthritis. Rheumatology (Oxford).

[6] Malaviya AP, Ostor AJK (2011). Early diagnosis crucial in ankylosing spondylitis. Practitioner.

[7] van der Linden MPM, le Cessie S, Raza K (2010). Long-term impact of delay in assessment of patients with early arthritis. Arthritis Rheum.

[8] (2025). Spending on health care up by 8.1 percent in 2024. Centraal Bureau voor de Statistiek.

[9] van der Waal JM, Bot SDM, Terwee CB, van der Windt D, Bouter LM, Dekker J (2003). Determinants of the clinical course of musculoskeletal complaints in general practice: design of a cohort study. BMC Musculoskelet Disord.

[10] Stack RJ, Nightingale P, Jinks C (2019). Delays between the onset of symptoms and first rheumatology consultation in patients with rheumatoid arthritis in the UK: an observational study. BMJ Open.

[11] Raza K, Stack R, Kumar K (2011). Delays in assessment of patients with rheumatoid arthritis: variations across Europe. Ann Rheum Dis.

[12] Knitza J, Knevel R, Raza K (2020). Toward earlier diagnosis using combined eHealth tools in rheumatology: the joint pain assessment scoring tool (JPAST) project. JMIR Mhealth Uhealth.

[13] (1998). Act of 26 February 1998 on medical-scientific research involving human subjects (Medical Research Involving Human Subjects Act, WMO). Dutch Official Gazette.

[14] Snoek J, Larochelle H, Adams RP (2012). Practical Bayesian optimization of machine learning algorithms. arXiv.

[15] Chen T, Guestrin C, Krishnapuram B, Shah M KDD ’16: Proceedings of the 22nd ACM SIGKDD International Conference on Knowledge Discovery and Data Mining.

[16] Grinsztajn L, Oyallon E, Varoquaux G, Koyejo S, Mohamed S, Agarwal A, Belgrave D, Cho K, Oh A (2022). NIPS ’22: Proceedings of the 36th International Conference on Neural Information Processing Systems.

[17] Roth AE (1988). The Shapley Value: Essays in Honor of Lloyd S Shapley.

[18] Miloslavsky EM, Marston B (2022). The challenge of addressing the rheumatology workforce shortage. J Rheumatol.

[19] Graydon SL, Thompson AE (2008). Triage of referrals to an outpatient rheumatology clinic: analysis of referral information and triage. J Rheumatol.

[20] Hügle M, Omoumi P, van Laar JM, Boedecker J, Hügle T (2020). Applied machine learning and artificial intelligence in rheumatology. Rheumatol Adv Pract.

[21] Knitza J, Mohn J, Bergmann C (2021). Accuracy, patient-perceived usability, and acceptance of two symptom checkers (Ada and Rheport) in rheumatology: interim results from a randomized controlled crossover trial. Arthritis Res Ther.

[22] Brorsson S, Hilliges M, Sollerman C, Nilsdotter A (2009). A six-week hand exercise programme improves strength and hand function in patients with rheumatoid arthritis. J Rehabil Med.

[23] Baillet A, Payraud E, Niderprim VA (2009). A dynamic exercise programme to improve patients’ disability in rheumatoid arthritis: a prospective randomized controlled trial. Rheumatology (Oxford).

[24] Crofford LJ (2013). Use of NSAIDs in treating patients with arthritis. Arthritis Res Ther.

[25] Salvarani C, Vlachonikolis IG, van der Heijde DM (2001). Musculoskeletal manifestations in a population-based cohort of inflammatory bowel disease patients. Scand J Gastroenterol.

[26] Chinn S, Caldwell W, Gritsenko K (2016). Fibromyalgia pathogenesis and treatment options update. Curr Pain Headache Rep.

[27] Wang B, Li W, Bradlow A, Bazuaye E, Chan ATY (2023). Improving triaging from primary care into secondary care using heterogeneous data-driven hybrid machine learning. Decis Support Syst.

[28] Maarseveen TD, Glas HK, Veris-van Dieren J, van den Akker E, Knevel R (2025). Improving musculoskeletal care with AI enhanced triage through data driven screening of referral letters. NPJ Digit Med.

[29] Schäfer A, Kovacs MS, Nigg A, Feuchtenberger M (2024). Patient-reported outcomes of depression and fibromyalgia symptoms do not predict non-inflammatory versus inflammatory diagnoses at initial rheumatology consultation. Healthcare (Basel).

[30] Gomon G, Raffray M, Pérez - Sancristóbal I (2025). POS0433 comparison of musculoskeletal referral pathways across EUROPE. Ann Rheum Dis.

[31] Krusche M, Callhoff J, Knitza J, Ruffer N (2024). Diagnostic accuracy of a large language model in rheumatology: comparison of physician and ChatGPT-4. Rheumatol Int.

[32] Wu J, Dong H, Li Z (2024). A hybrid framework with large language models for rare disease phenotyping. BMC Med Inform Decis Mak.

[33] Shyr C, Hu Y, Bastarache L (2024). Identifying and extracting rare diseases and their phenotypes with large language models. J Healthc Inform Res.

[34] Soteriades ES, Psalta L, Leka S, Spanoudis G (2019). Occupational stress and musculoskeletal symptoms in firefighters. Int J Occup Med Environ Health.

[35] Kivimäki M, Leino-Arjas P, Virtanen M (2004). Work stress and incidence of newly diagnosed fibromyalgia: prospective cohort study. J Psychosom Res.

[36] Lewis KL, Battaglia PJ (2019). Differentiating bilateral symptomatic hand osteoarthritis from rheumatoid arthritis using sonography when clinical and radiographic features are nonspecific: a case report. J Chiropr Med.

[37] Maarseveen TD FRYQ. Github.

