# Supplementary material for: Evaluating the Accuracy of the Frysian Questionnaire for Differentiation of Musculoskeletal Complaints for Triage of Musculoskeletal Diseases: Algorithm Development and Validation Study
Source: JMIR Med Inform. 2025 Nov 17;13:e77345. doi: 10.2196/77345 (PMC12622856; doi:10.2196/77345)
Supplement: Multimedia Appendix 1 [file medinform-v13-e77345-s001.docx]

**VRAGENLIJST**

1. **Mijn klachten bestaan voornamelijk uit pijn. Eens/oneens**

Indien oneens, ga door naar vraag 2:

1. *Ik word ‘s nacht wakker van de pijn…………………………………………..…….. Eens/oneens*

*2. Ik word vooral tegen de ochtend wakker van de pijn……………………………. Eens/oneens*

*3. De pijn is de hele dag aanwezig…………………………………………………………… Eens/oneens*

*4. De pijn wordt beter bij bewegen………………………………………………………….. Eens/oneens*

*5. De pijn wordt beter door rust………………………………………………………………. Eens/oneens*

*6. Bij geringe aanraking is het heel pijnlijk………………………………………………. Eens/oneens*

*7. Na aanraken houdt de pijn nog meer dan een half uur aan………………… Eens/oneens*

*8. Bij stoten is de plek pijnlijk………………………………………………………………….. Eens/oneens*

*9. Bij omdraaien in bed heb ik pijn aan de buitenkant van mijn heupen…. Eens/oneens*

*10. Ik heb pijn sinds:*

*0-6 weken / 6 -16 weken / 4-12 maanden / langer dan 1 jaar/ langer dan 10 jaar*

*11. Een ontstekingsremmer als ibuprofen of diclofenac helpt goed……….. Eens/oneens/*

*niet gebruikt*

1. **Mijn klachten bestaan ook uit stijfheid. Eens/oneens**

Indien oneens, ga door naar vraag 3:

1. *De stijfheid is vooral in de ochtend………………….………………………….…… Eens/oneens*

*2. De stijfheid is vooral na rust……………………………………………………………. Eens/oneens*

*3. De stijfheid duurt minder dan 30 minuten na het wakker worden……. Eens/oneens*

*4. De stijfheid duurt langer dan 45 minuten na het wakker worden…….. Eens/oneens*

*5. De stijfheid wordt beter na rust………………………………………………………. Eens/oneens*

*6. De stijfheid wordt beter bij bewegen………………………………………………. Eens/oneens*

*7. De stijfheid is acuut ontstaan en zit vooral in schouders en heupen…. Eens/oneens*

*8. Ik heb stijfheid sinds:*

*0-6 weken / 6 -16 weken / 4-12 maanden / langer dan 1 jaar/ langer dan 10 jaar*

*9. Een ontstekingsremmer als ibuprofen of diclofenac helpt goed……….. Eens/oneens/*

*niet gebruikt*

1. **Door mijn klachten:**

1. *Functioneer ik minder goed in mijn werk…………………………………………. Eens/oneens*

*2. Heb ik mij ziek gemeld op het werk…………………………………………………. Eens/oneens*

*3. Kan ik mijn hobby’s minder goed uitvoeren…………………………………….. Eens/oneens*

*4. Kan ik minder goed mijn huishouden doen……………………………………… Eens/oneens*

*5. Ben ik somber……………….………………………………………………………………… Eens/oneens*

*6. De somberheid was voor de klachten niet aanwezig…………………….... Eens/oneens*

1. **Geef aan op het plaatje (bv met een kruisje) waar de klachten zich bevinden:**


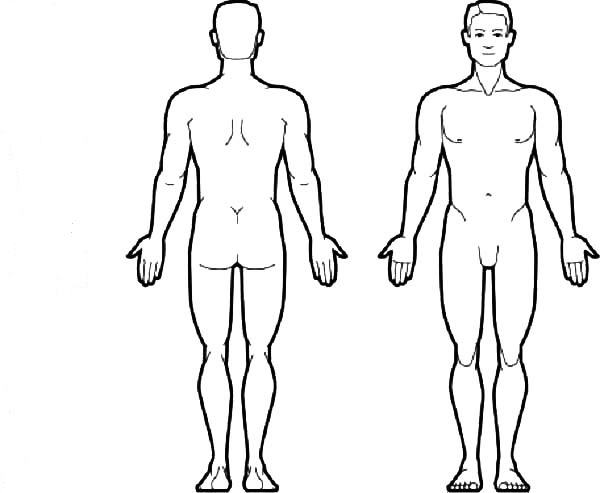


1. **Met bovenstaande plaatsen wordt bedoeld, waar een cirkel omheen staat.**

De bovenstaande plaatsen zijn weleens gezwollen………………………………………………….. Eens/oneens

De bovenstaande plaatsen zijn altijd gezwollen………………………………………………………. Eens/oneens

Als er ook of vooral rugklachten zijn (Indien niet aanwezig, ga door naar vraag 6):

1. *De rug betreft een pijnlijk gevoel*………..………………………………………………. *Eens/oneens*

*2. De rug betreft een stijf gevoel………………………………………………………. ….... Eens/oneens*

*3. De rugklachten worden beter bij bewegen………………………………………….. Eens/oneens*

*4. Ik heb dove gevoelens in de benen………………………………………………......... Eens/oneens*

*5. Ik heb uitstralende pijn naar/in de benen……………………………………………. Eens/oneens*

*6. De klachten in de benen worden erger bij hoesten, niezen of persen….. Eens/oneens*

1. **Ik heb last van:**

**1. Psoriasis ………………………………………………………………………………………………….. Eens/oneens**

Indien eens:

*Ik ben hiervoor onder behandeling van de dermatoloog/huisarts……. Eens/oneens*

**2. Colitis ulcerosa/De ziekte van Crohn (ontstekingen in de darmen)…………… Eens/oneens**

Indien eens:

*Ik ben hiervoor onder behandeling van de MDL-arts/internist………… Eens/oneens*

**3. Oogontstekingen……………………………………………………………………………………… Eens/oneens**

Indien eens:

*Ik ben hiervoor onder behandeling van de oogarts/huisarts……………. Eens/oneens*

**4. Fenomeen van Raynaud (vingertoppen in de kou pijnlijk en wit, blauw, rood).Eens/oneens**

Indien eens:

*Ik ben hiervoor onder behandeling van de dermatoloog/huisarts……. Eens/oneens*

**5. Sarcoïdose………………………………………………………………………………………………..** **Eens/Oneens**

*Indien eens:*

*Ik ben hiervoor onder behandeling van de longarts(of andere specialist)/huisarts*

*Eens/oneens*

**6. Zonneallergie (jeuk/bultjes op de huid waar zon op schijnt)…………………….. Eens/oneens**

Indien eens:

*Ik ben hiervoor behandeling van de dermatoloog/huisarts……………… Eens/oneens*

**7. Droge ogen/mond …………………………………………………………………………………….. Eens/oneens**

Indien eens:

*Ik heb last van droge hoest, altijd drinken bij eten, meer gaatjes in het gebit*

*Ik heb last van zandgevoel in de ogen*

**8. Eten wat niet wil zakken Eens/oneens**

Indien eens:

*Dit is voor vast eten……………………………………………………………………….. Eens/oneens*

*Dit is voor vloeibaar eten…………………………………………………………… Eens/oneens*

**9. Aften/blaren in de mond Eens/oneens**

Indien eens:

*Dit is meer dan 2x per maand……………………………………………………….. Eens/oneens*

**10. Koorts…………………………………………………………………………………………………...... Eens/oneens**

Indien eens:

Wat is de temperatuur die is gemeten?

**11. Afvallen (zonder aanpassen van dieet)…………………………………………………….. Eens/oneens**

Indien eens:

Hoeveel bent u afgevallen en in hoeveel tijd?

*De eetlust is minder…………………………………………………………………………. Eens/oneens*

**12. Pijn op de borst……………………………………………………………………………………….. Eens/oneens**

Indien eens:

*Dit is vooral bij inspanning…………………………………………………………………Eens/oneens*

*Dit is vooral een drukkend gevoel…………………………………………………….. Eens/oneens*

**13. Kortademigheidsklachten………………………………………………………………………. Eens/oneens**

Indien eens:

*Dit is vooral bij inspanning………………………………………………………………. Eens/oneens*

*Dit is vooral bij plat liggen……………………………………………………………….. Eens/oneens*

**15. Veranderd ontlastingspatroon……………………………………………………………………… Eens/oneens**

Indien eens:

*Nu meer verstopping……………………………………………………………………….. Eens/oneens*

*Nu vaker ontlasting…………………………………………………………………………. Eens/oneens*

*Nu meer dunne ontlasting……………………………………………………………….. Eens/oneens*

*Nu bloed bij de ontlasting……………………………………………………………….. Eens/oneens*

*Nu slijm bij de ontlasting……………………………………………………………..... Eens/oneens*

1. **Geef aan of u het eens of oneens bent met onderstaande stellingen**

1. Ik heb zwaar werk……………………………………………………………………………………..………… Eens/oneens

2. Ik doe thuis het huishouden……………………………………………………………………..…………. Eens/oneens

3. Ik kan mijn werk uitvoeren zoals ik wil………………………………………………………..………. Eens/oneens

4. Ik kan mijn hobby’s doen zoals ik wil……………………………………………………..……………. Eens/oneens

5. Ik kan mijn huishouden uitvoeren zoals ik wil………………………………………..……………. Eens/oneens

6. Ik was heel lenig…………………………………………………………………………………..……………… Eens/oneens

7. Ik ben nog steeds heel lenig………………………………………………………………….…….………. Eens/oneens

8. Ik kan ’s nachts goed slapen…………………………………………………………………..……………. Eens/oneens

9. Ik word bijna nooit moe wakker…………………………………………………………..……………… Eens/oneens

10. Overdag wil ik het liefste nog een dutje doen……………………………………………………. Eens/oneens

11. Ik kan geen “nee” zeggen als iemand mij om een gunst vraagt……………………….... Eens/oneens

12. Ik weet goed wat ik kan en wat ik niet kan……………………………………………………..…. Eens/oneens

13. Ik ga bijna nooit over mijn grenzen heen…………………………………………………………… Eens/oneens

14. Ik heb een hoge pijngrens…………………………………………………………………………………. Eens/oneens

15. Ik sta altijd voor anderen klaar………………………………………………………………………….. Eens/oneens

16. Ik heb nog voldoende tijd voor mezelf (bv hobby’s)………………………………………….. Eens/oneens

17. Ik ben perfectionistisch……………………………………………………………………………………… Eens/oneens

1. **Zet op onderstaande lijnen een kruisje.**

***Dit is een voorbeeld:***

*Stel dat u erg veel last heeft van de pijn, zet u een kruisje in de buurt van veel pijn.*

________________________________________________________________**x**_______

Geen pijn Veel pijn

*Pijn zoals die afgelopen week was:*

Niet erg Heel erg

Ik voel me over het algemeen:

Goed Slecht

Moeheid:

Geen Heel erg

1. **OPEN VRAGEN**

1. Komen er in de familie ziekten/aandoeningen voor zoals reumatische aandoeningen? Zo ja, welke aandoening en bij wie?

2. Kunt u aangeven wat er is gebeurd met u of in uw omgeving voordat de klachten begonnen?

3. Kunt u iets vertellen over uw huidige/voorgaande werk?

4. Kunt u iets vertellen over uw hobby’s/werkzaamheden?

5. Kunt u iets vertellen over uw gezinssamenstelling?

6. Wat denkt u zelf dat er aan de hand is?

7. Maakt u zich ergens zorgen over? Indien ja, zo mogelijk nader specificeren.

8. Zijn er elders al onderzoeken verricht? Zo ja, welke en waar?

Weet u ook de uitkomst? Zo ja, welke was dat?

9. Gebruikt u hulpmiddelen, ter verbetering van klachten danwel om u zelf te kunnen redden met de dagelijkse bezigheden? Zo ja, welke?
