# Supplementary material for: Evaluating the Accuracy of the Frysian Questionnaire for Differentiation of Musculoskeletal Complaints for Triage of Musculoskeletal Diseases: Algorithm Development and Validation Study
Source: JMIR Med Inform. 2025 Nov 17;13:e77345. doi: 10.2196/77345 (PMC12622856; doi:10.2196/77345)
Supplement: Multimedia Appendix 2 [file medinform-v13-e77345-s002.docx]

***Multimedia Appendix 2: Table comprising the English translation of all original Dutch questions from the Frysian questionnaire***

| ***Question (English translation)*** | ***Original Dutch question*** |
| --- | --- |
| 1.1 I wake up at night due to the pain | 1.1 Ik word ‘s nachts wakker van de pijn |
| 1.2 I mostly wake up in the morning due to the pain | 1.2 Ik word vooral tegen de ochtend wakker van de pijn |
| 1.3 The pain is present all day | 1.3 De pijn is de hele dag aanwezig |
| 1.4 The pain improves with movement | 1.4 De pijn wordt beter bij bewegen |
| 1.5 The pain improves with rest | 1.5 De pijn wordt beter door rust |
| 1.6 Even light touch is very painful | 1.6 Bij geringe aanraking is het al heel pijnlijk |
| 1.7 After touching, the pain persists for more than half an hour | 1.7 Na aanraken houdt de pijn nog meer dan een half uur aan |
| 1.8 The spot is painful when bumped | 1.8 Bij stoten is de plek pijnlijk |
| 1.9 When turning over in bed, I have pain on the outside of my hips | 1.9 Bij omdraaien in bed heb ik pijn aan de buitenkant van mijn heupen |
| 1.10 I have pain since: | 1.10 Ik heb pijn sinds: |
| 1.11 Anti-inflammatory like ibuprofen/diclofenac works well against pain | 1.11 Een ontstekingsremmer als ibuprofen of diclofenac helpt goed (tegen pijn) |
| 2.1 The stiffness is especially in the morning | 2.1 De stijfheid is vooral in de ochtend |
| 2.2 The stiffness is especially after rest | 2.2 De stijfheid is vooral na rust |
| 2.3 The stiffness lasts less than 30 minutes after waking up | 2.3 De stijfheid duurt minder dan 30 minuten na het wakker worden |
| 2.4 The stiffness lasts longer than 45 minutes after waking up | 2.4 De stijfheid duurt langer dan 45 minuten na het wakker worden |
| 2.5 The stiffness improves with rest | 2.5 De stijfheid wordt beter na rust |
| 2.6 The stiffness improves with movement | 2.6 De stijfheid wordt beter bij bewegen |
| 2.7 The stiffness started suddenly and is mainly in the shoulders and hips | 2.7 De stijfheid is acuut ontstaan en zit vooral in schouders en heupen |
| 2.8 I have had stiffness since | 2.8 Ik heb stijfheid sinds: |
| 2.9 Anti-inflammatory like ibuprofen/diclofenac works well against stiffness | 2.9 Een ontstekingsremmer als ibuprofen of diclofenac helpt goed |
| 3.1 Due to my complaints: I perform less well at work | 3.1 Door mijn klachten: Functioneer ik minder goed in mijn werk |
| 3.2 Due to my complaints: I have reported sick at work | 3.2 Door mijn klachten: Heb ik mij ziek gemeld op het werk |
| 3.3 Due to my complaints: I can perform my hobbies less well | 3.3 Door mijn klachten: Kan ik mijn hobby’s minder goed uitvoeren |
| 3.4 Due to my complaints: I can do my household chores less well | 3.4 Door mijn klachten: Kan ik minder goed mijn huishouden doen |
| 3.5 Due to my complaints: I am feeling down | 3.5 Door mijn klachten: Ben ik somber |
| 3.6 Due to my complaints: (The feeling of sadness was not present before the complaints) | 3.6 Door mijn klachten: (De somberheid was voor de klachten niet aanwezig) |
| 4.1 I suffer from psoriasis | 6.1 Ik heb last van psoriasis |
| 4.2 I suffer from ulcerative colitis/Crohn's disease (inflammation in the intestines) | 6.2 Ik heb last van Colitis ulcerosa/De ziekte van Crohn (ontstekingen in de darmen) |
| 4.3 I suffer from eye inflammations | 6.3 Ik heb last van oogontstekingen |
| 4.4 I suffer from Raynaud's phenomenon (fingertips become painful and turn white/blue in the cold, then turn red again) | 6.4 Ik heb last van Fenomeen van Raynaud (vingertoppen worden bij kou pijnlijk en wit/ blauw en kleuren weer rood bij) |
| 4.5 I suffer from sarcoidosis | 6.5 Ik heb last van Sarcoïdose |
| 4.6 I suffer from sun allergy (itching/bumps on the skin where the sun has shone) | 6.6 Ik heb last van zonneallergie (jeuk/bultjes op de huid waarop de zon heeft geschenen) |
| 4.7 I suffer from dry eyes/mouth | 6.7 Ik heb last van droge ogen/mond |
| 4.8 I suffer from food that won’t go down (in the esophagus) | 6.8 Ik heb last van eten dat niet wil zakken (in de slokdarm) |
| 4.9 I suffer from canker sores/blisters in the mouth | 6.9 Ik heb last van aften/blaren in de mond |
| 4.10 I suffer from fever | 6.10 Ik heb last van koorts |
| 4.11.1 How much weight have you lost? | 6.11.1 Hoeveel ben u afgevallen? |
| 4.12 I suffer from chest pain | 6.12 Ik heb last van pijn op de borst |
| 4.13 I suffer from shortness of breath | 6.13 Ik heb last van kortademigheidsklachten |
| 4.15 I suffer from a changed bowel movement pattern | 6.15 Ik heb last van een veranderd ontlastingspatroon |
| 5.1 I do heavy work | 7.1 Ik heb zwaar werk |
| 5.2 I do the household chores at home | 7.2 Ik doe thuis het huishouden |
| 5.3 I can perform my work the way I want | 7.3 Ik kan mijn werk uitvoeren zoals ik wil |
| 5.4 I can perform my hobbies the way I want | 7.4 Ik kan mijn hobby's uitvoeren zoals ik wil |
| 5.5 I can do my household chores the way I want | 7.5 Ik kan mijn huishouden uitvoeren zoals ik wil |
| 5.6 I was very flexible | 7.6 Ik was heel lenig |
| 5.7 I am still very flexible | 7.7 Ik ben nog steeds heel lenig |
| 5.8 I sleep well at night | 7.8 Ik kan ‘s nachts goed slapen |
| 5.9 I almost never wake up feeling tired | 7.9 Ik word bijna nooit moe wakker |
| 5.10 During the day, I’d most like to take a nap | 7.10 Overdag wil ik het liefste nog een dutje doen |
| 5.11 I can’t say "no" when someone asks me for a favor | 7.11 Ik kan geen ‘nee’ zeggen als iemand mij om een gunst vraagt |
| 5.12 I know well what I can and cannot do | 7.12 Ik weet goed wat ik kan en wat ik niet kan |
| 5.13 I almost never go beyond my limits | 7.13 Ik ga bijna nooit over mijn grenzen heen |
| 5.14 I have a high pain threshold | 7.14 Ik heb een hoge pijngrens |
| 5.15 I am always there for others | 7.15 Ik sta altijd voor anderen klaar |
| 5.16 I still have enough time for myself (e.g., hobbies) | 7.16 Ik heb nog voldoende tijd voor mezelf (bv hobby’s) |
| 5.17 I am perfectionistic | 7.17 Ik ben perfectionistisch |
| 6.1 Pain like it was last week | 8.1 Pijn zoals die afgelopen week was |
| 6.2 In general I feel: | 8.2 Ik voel me over het algemeen: |
| 6.3 Fatigue | 8.3 Moeheid: |
| 7.1 Are there any diseases/conditions in the family, such as rheumatic conditions? If yes, which condition and in whom? | 9.1 Komen er in de familie ziekten/aandoeningen voor zoals reumatische aandoeningen? Zo ja, welke aandoening en bij wie? |
| 7.2 Can you indicate what happened to you or in your environment before the complaints started? | 9.2 Kunt u aangeven wat er is gebeurd met u of in uw omgeving voordat de klachten begonnen? |
| 7.3 Can you describe your current/previous work? | 9.3 Kunt u iets vertellen over uw huidige/voorgaande werk? |
| 7.4 Can you describe your hobbies/activities? | 9.4 Kunt u iets vertellen over uw hobby’s/werkzaamheden? |
| 7.5 Can you describe your family composition? | 9.5 Kunt u iets vertellen over uw gezinssamenstelling? |
| 7.6 What do you think is going on? | 9.6 Wat denkt u zelf dat er aan de hand is? |
| 7.7 Are you worried about anything? If yes, please specify if possible. | 9.7 Maakt u zich ergens zorgen over? Indien ja, zo mogelijk nader specificeren. |
| 7.8 Have any investigations been carried out elsewhere? If yes, which ones and where? Do you also know the outcome? If yes, what was it? | 9.8 Zijn er elders al onderzoeken verricht? Zo ja, welke en waar? Weet u ook de uitkomst? Zo ja, welke was dat? |
| 7.9 Do you use any aids to improve the complaints or to manage daily activities? If yes, which aids? | 9.9 Gebruikt u hulpmiddelen, ter verbetering van de klachten dan wel om u zelf te kunnen redden met dagelijkse bezigheden? Zo ja, welke hulpmiddelen? |
| Sex | Geslacht |
