# Supplementary material for: Evaluating the Accuracy of the Frysian Questionnaire for Differentiation of Musculoskeletal Complaints for Triage of Musculoskeletal Diseases: Algorithm Development and Validation Study
Source: JMIR Med Inform. 2025 Nov 17;13:e77345. doi: 10.2196/77345 (PMC12622856; doi:10.2196/77345)
Supplement: Multimedia Appendix 3 [file medinform-v13-e77345-s003.pdf]

Multimedia Appendix 3 : Supplementary Results

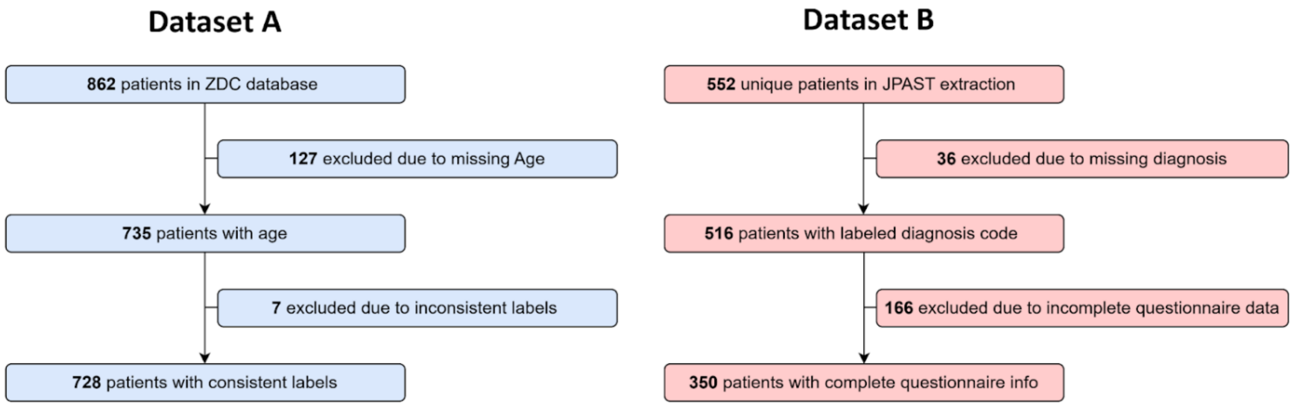

Figure S1. Patient selection flow for both cohorts.

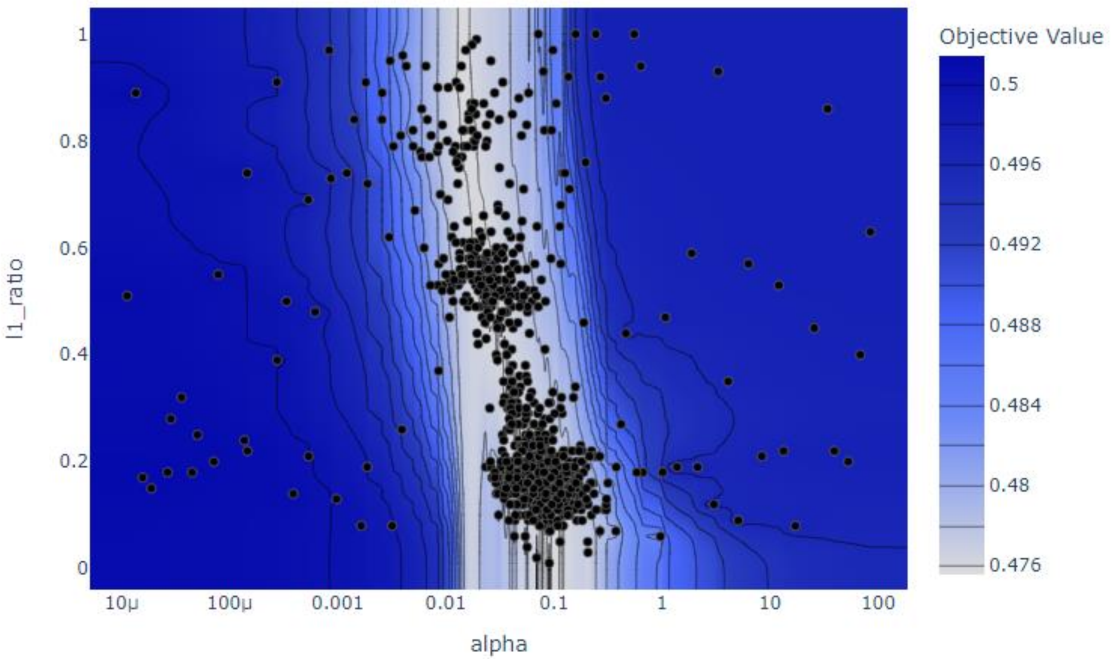

Figure S2. Contour plot showing the most optimal configurations within the parametric space for the elastic net. Where the objective value is to minimize root mean squared error for the task of differentiating IRDs from non-IRDs.

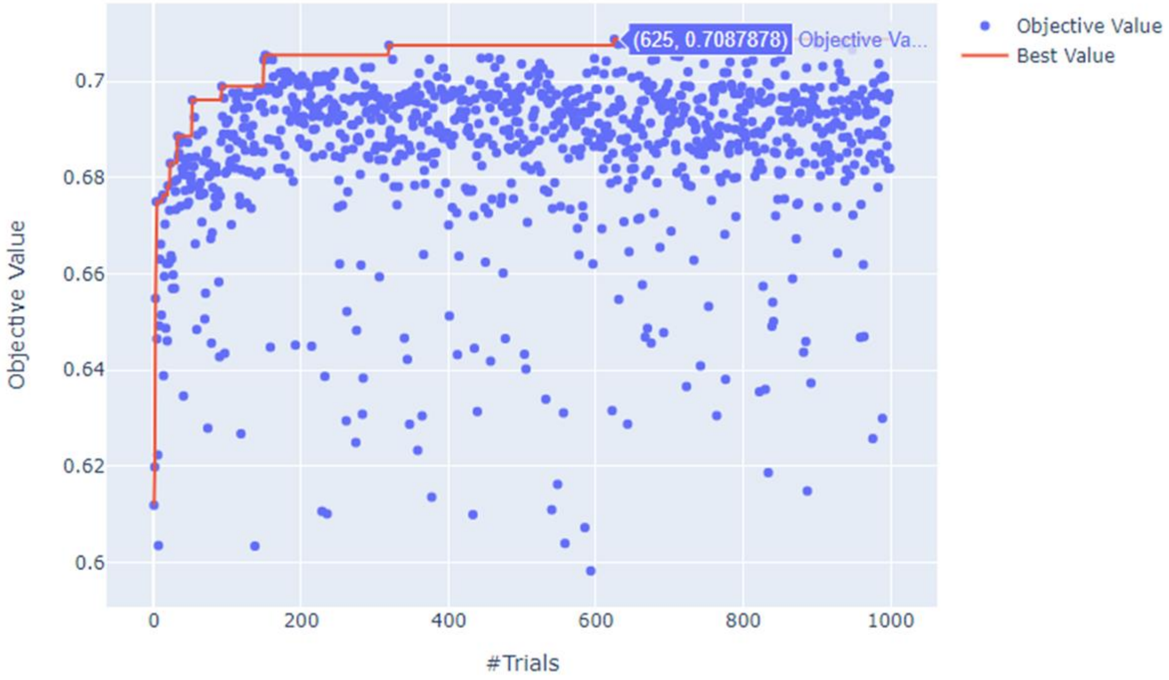

Figure S3. Optimization curve of the gradient boosting algorithm showing the best AUC-ROC values obtained during 1000 hyperparameter tuning iterations in the training partition of Dataset A.

Sens	*Spec*	*PPV*	*NPV*	*Acc*
0.15	0.96	0.77	0.57	0.59
0.37	0.85	0.68	0.61	0.63
0.54	0.81	0.71	0.67	0.68
0.76	0.49	0.56	0.71	0.62
0.88	0.38	0.55	0.79	0.61
0.97	0.18	0.50	0.88	0.54

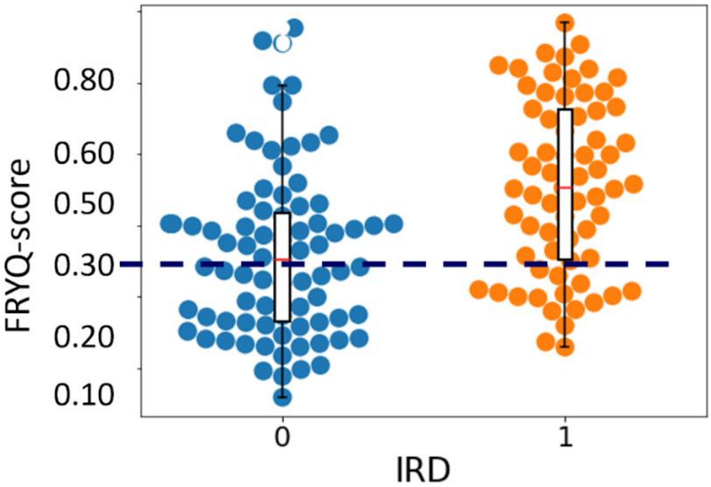

Figure S4. Swarm plot illustrating the model-derived probability scores for having Inflammatory Rheumatic disease, for patients with- (in orange) and without (in blue) an IRD. The dotted lines display the optimal cutoff. Where Sens= sensitivity, Spec= specificity; PPV= positive predictive value; NPV= negative predictive value; Acc= accuracy.

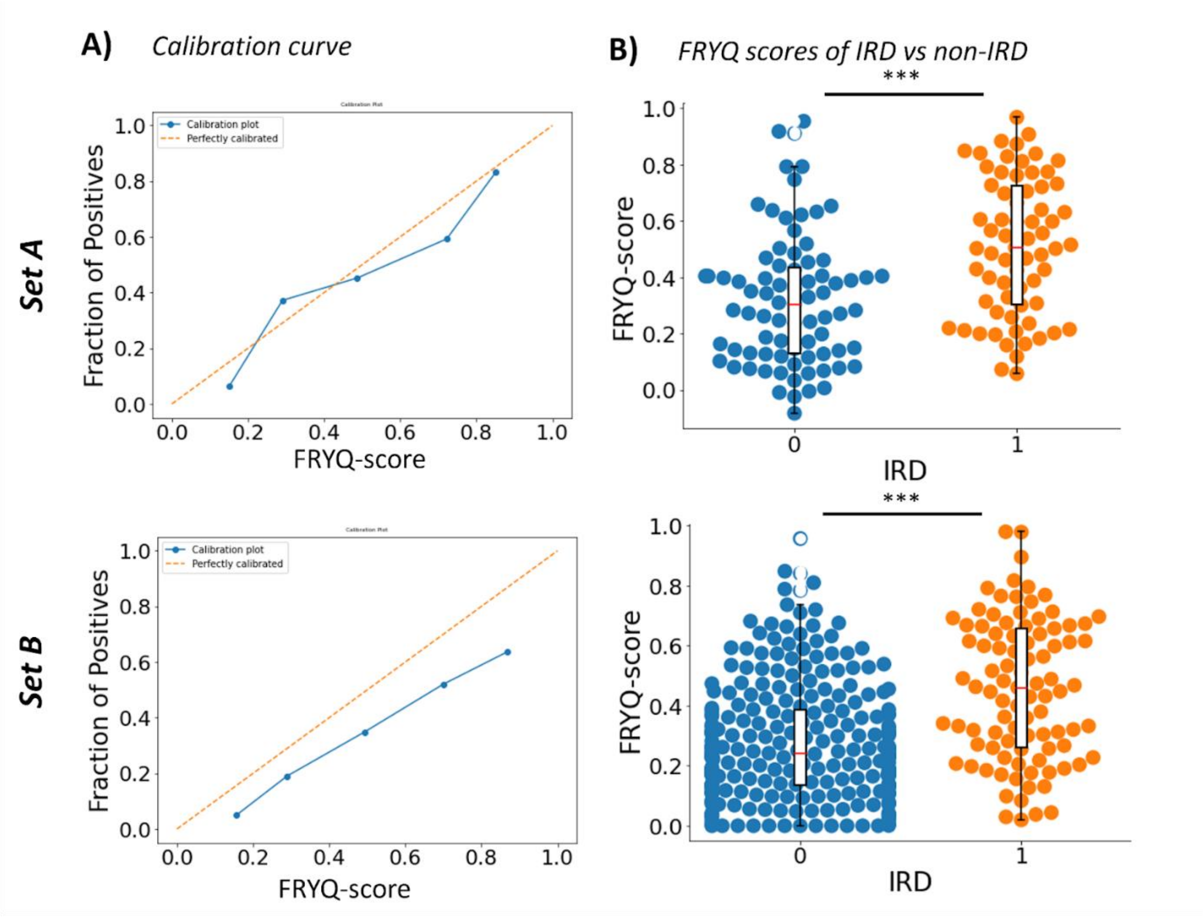

Figure S5. Calibration of the FRYQ-questionnaire: (A) showing the relationship between predicted FRYQ scores and the observed proportion of inflammatory rheumatic disease (IRD) cases and (B) Swarm plot displaying the distribution of true positives across the full range of predicted probabilities.

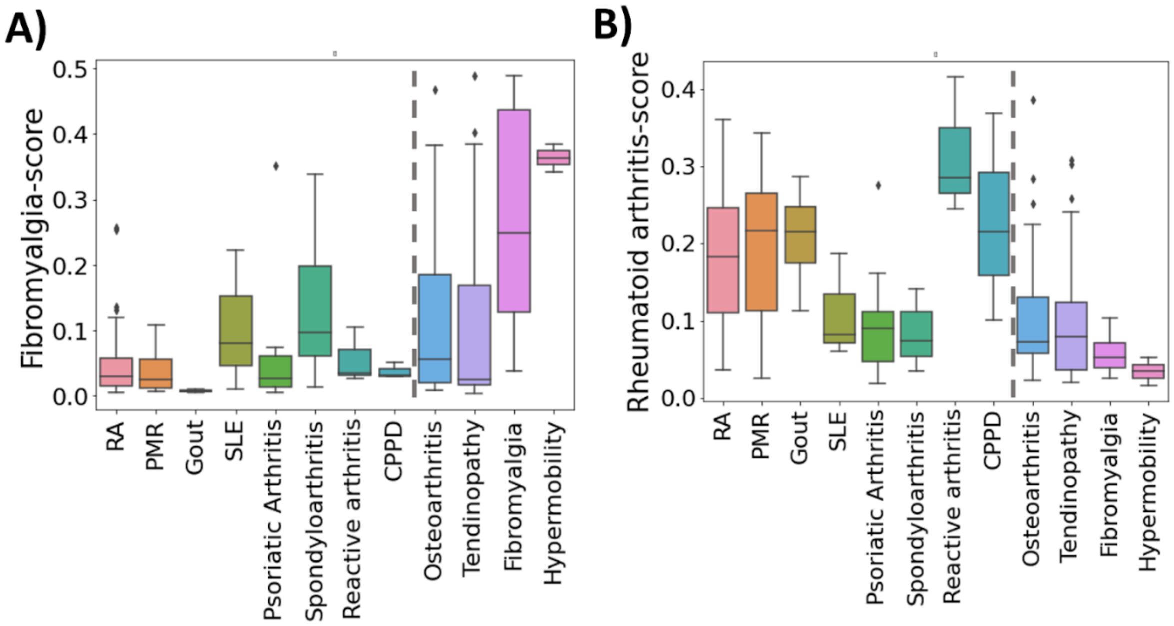

Figure S6: The probabilities of the specialized models expressed for the different disease entities: (A) probabilities of the Fibromyalgia model. (B) probabilities of the Rheumatoid arthritis model

Table S1: Final set of questions selected by the elastic net, with corresponding coefficients

Questions	coef
1.4 The pain improves with movement	0.06173822962352353
1.7 After touching, the pain persists for more than half an hour	-0.0034399406935199414
1.9 When turning over in bed, I have pain on the outside of my hips	-0.014461543130268536
1.10 I have pain since:	-0.07245737117027902
1.11 Anti-inflammatory like ibuprofen/diclofenac works well against pain	0.009304200060137349
2.4 The stiffness lasts longer than 45 minutes after waking up	0.002175954701317089
2.8 I have had stiffness since	-0.036534824786869445
2.9 Anti-inflammatory like ibuprofen/diclofenac works well against stiffness	-0.014632279999653948
3.6 Due to my complaints: (The feeling of sadness was not present before the complaints)	-0.023234779924971062
4.2 I suffer from ulcerative colitis/Crohn's disease (inflammation in the intestines)	0.0358558508437731
4.8 I suffer from food that won’t go down (in the esophagus)	0.004274309887613922
4.12 I suffer from chest pain	-0.015121484582639205
4.13 I suffer from shortness of breath	0.004411989323252082
5.1 I do heavy work	-0.018178995628625794
5.3 I can perform my work the way I want	0.017142617721693294
5.5 I can do my household chores the way I want	<0.0
5.6 I was very flexible	-0.0
5.7 I am still very flexible	0.0
5.8 I sleep well at night	0.0
5.9 I almost never wake up feeling tired	0.026404676931562437
5.13 I almost never go beyond my limits	0.02177255863190188

5.14 I have a high pain threshold	0.0
5.15 I am always there for others	-0.0
5.17 I am perfectionistic	-0.004439285355490346
6.1 Pain like it was last week	-0.0034529241771348675
6.2 In general I feel:	-0.007810175558100415
6.3 Fatigue	-0.004148002084181895
Sex	-0.08545925209944166

Table S2. Resulting confusion tables of IRD classification according to FRYQ on the validation set (Dataset A) and the replication set (Dataset B) as chosen cut-offs.

**Dataset A validation (threshold=0.30)**			**Dataset B replication (threshold=0.30)**		
**Reference**	*Non-IRD*	*IRD*	**Reference**	*Non-IRD*	*IRD*
**predictions**			**predictions**		
Non-IRD	39 (TN)	40 (FP)	*Non-IRD*	155 (TN)	102 (FP)
IRD	16 (FN)	51 (TP)	*IRD*	27(FN)	66 (TP)
**Metrics**					
Sens		0.76	*Sens*		0.71
Spec		0.49	*Spec*		0.59
PPV		0.56	*PPV*		0.39
NPV		0.71	*NPV*		0.85
Accuracy		0.62	*Accuracy*		0.62

TN = True negative, FP = False positive, FN = False negative, TP = True positive, Sens = Sensitivity, Spec = Specificity, PPV = Positive Predictive value, NPV = Negative Predictive Value, Acc = Accuracy,
